# Dietary Habits of Type 2 Diabetes Patients: Variety and Frequency of Food Intake

**DOI:** 10.1155/2016/7987395

**Published:** 2016-12-22

**Authors:** Senadheera Pathirannehelage Anuruddhika Subhashinie Senadheera, Sagarika Ekanayake, Chandanie Wanigatunge

**Affiliations:** ^1^Department of Biochemistry, Faculty of Medical Sciences, University of Sri Jayewardenepura, Nugegoda, Sri Lanka; ^2^Department of Pharmacology, Faculty of Medical Sciences, University of Sri Jayewardenepura, Nugegoda, Sri Lanka

## Abstract

The objective of the present study was to observe the dietary patterns and food frequencies of type 2 diabetes patients attending the clinics of the Family Practice Center of the University of Sri Jayewardenepura, located in a highly urbanized area in Sri Lanka. An interviewer administered questionnaire based cross-sectional study was conducted among randomly selected 100 type 2 diabetes patients [age 35–70 years; mean age 55 ± 9 (males = 44; females = 56)]. The data were analyzed by SPSS version 18.0 software. Vegetables, fatty foods, and poultry consumption were in accordance with the national guidelines. A significant percentage (45.5%) consumed rice mixed meals for all three meals and only 67% consumed fruits at least once a day. Majority (71%) consumed full-cream milk and sugar intake (77%) was in accordance with the guidelines. Noncaloric sweetener usage was nonexistent. Daily green leafy vegetable intake and the quantity consumed were inadequate to obtain beneficial effects. From the study population, 44% [females 50%; males 36%] of the patients were either overweight or obese. However, only 60% of those patients accepted that they were either overweight or obese. Only 14% exercised daily while 69% never exercised. Study revealed the importance of educating patients with type 2 diabetes on dietary changes and more importantly the involvement in regular physical exercises.

## 1. Introduction

Diabetes mellitus is a group of metabolic diseases characterized by hyperglycemia that result from defects in insulin secretion, action, or both. Improved glycaemic control among type 2 diabetes patients is vital in preventing micro- and macrovascular complications. Type 2 diabetes has become a severe global health threat with increasing incidence in Asian countries [1]. Sri Lanka is at high risk of diabetes with one in five adults having either diabetes or prediabetes [2]. Several studies emphasize the importance of good dietary practices in management of diabetes mainly by reducing the body weight as a strong relationship between obesity and the upsurge of diabetes [3] is reported. Management is advocated through reduction of sugar and fat intake, increased dietary fibre consumption, and involvement in regular exercise [3, 4].

Urbanization has led many Sri Lankans towards a stressful, unhealthy life style, mainly altering their dietary patterns from the consumption of fresh, healthy food to more refined carbohydrates and high fat containing junk food and beverages. Thus unhealthy dietary habits and sedentary life style are among the leading causes of obesity and diabetes in Sri Lanka [5]. Almost 70% of a Sri Lankan study population exceeded the upper limit of the recommendations for starch intake [6] and consume 9 times more saturated fats compared to polyunsaturated fatty acids (PUFAs) [7]. More than 80% of the Sri Lankan adult population between the ages of 15 and 64 does not meet the recommended servings of fruits and vegetables. The recommended consumption of 5 portions of fruits and vegetables/day is practiced only by 3.5% of the Sri Lankan population [6]. The annual consumption of sugar by a Sri Lankan is around 30 kg with adults consuming over 3–5 portions of added sugar daily [6]. A recent research with adolescents of 17 years of age in 65 schools states that nearly 82% of the adolescents consume sugar-sweetened soft drinks once weekly or more often while 2% were daily consumers. Seventy-seven percent and 48% consumed sugar-sweetened carbonated drinks and sugar-sweetened fruit drinks once weekly or more often, respectively [8]. A more recent study revealed that although most of the Sri Lankan diabetes patients restrict sugar intake, they consume improper sugar alternatives (i.e., dates) in normal portion or in higher amounts without restriction. Even with the knowledge of adverse effects of sugar consumption, some of the study participants (18 out of 50) had difficulty in controlling the intake of sugar containing foods [9].

Further, a correlation between consumption of fast foods and noncommunicable diseases [10, 11] is reported. A study on the attitude of working women in Sri Lanka towards fast food consumption with reference to perceived taste, quality, nutrition value, convenience, and price found nonsignificant positive correlations with perceived taste and nutritional value [12]. Perceived convenience was the only significant factor for the increased fast food consumption among Sri Lankan working women [12].

The mean energy intake of type 2 diabetes patients in Sri Lanka was 1438 (SD 412) kcal/day. From the total energy intake, 68.1, 11.5, and 20.2% were from carbohydrate, protein, and fat, respectively. It was found that these patients consumed an energy restricted, high-carbohydrate, low fat diet compared to diabetes patients in Western countries [13]. Even though data are available on the dietary patterns and food frequencies of diabetes patients in Sri Lanka the information on macronutrient intake is scarce, except for the previously mentioned quantitative pilot study. Understanding the factors leading to improved glycaemic control would help professionals to identify major determinants of diabetes management. Therefore the objective of the present study was to identify the dietary patterns and food frequencies of type 2 diabetes patients attending the clinics of the Family Practice Center of the University of Sri Jayewardenepura, Nugegoda, Sri Lanka.

## 2. Methodology

A cross-sectional study was conducted among randomly selected type 2 diabetes patients (*n* = 100) between the ages of 35 and 70 years with a mean age of 55 ± 9 (males = 44; females = 56). Type 2 diabetes patients were identified from among those attending the clinic of the Family Practice Center of the University of Sri Jayewardenepura and employees of the University. Patients with severe medical and surgical complications were excluded.

Sample size was calculated based on the following equation and data:

(1) $$\begin{array}{c} Sample\;size=\frac{Z^{2}\times p\left(1-p\right)}{d^{2}}. \end{array}$$

*Z* is at 5% type 1 error (1.96), *p* is 3.5% (recommended consumption of fruits and vegetables/day by healthy Sri Lankan population [6]), *d* is absolute error/precision (0.05), and sample size is 1.96^2^ × 0.035 (1 − 0.035)/(0.05)^2^ = 52 (https://www.ncbi.nlm.nih.gov/pmc/articles/PMC3775042/).

### 2.1. Procedure

Study was an interviewer administered questionnaire based cross-sectional study. Data related to (i) sociodemographic data, weight, and height, (ii) awareness on overweight or obesity, (iii) consumption of main and other meals, (iv) consumption of green leaves, (v) consumption of sweetened foods, (vi) prevalence of diabetes related complications, and (vii) duration and type of physical exercises engaged in were collected using the questionnaire.

### 2.2. Ethical Approval

Ethical approval was obtained from the Ethics Review Committee of the Faculty of Medical Sciences, University of Sri Jayewardenepura (Approval number 632/12). Informed written consent was obtained from each volunteer prior to commencement of the study.

### 2.3. Data Analysis

The descriptive statistics were analyzed by SPSS version 18.0 software for Windows and Microsoft Excel (2007) and the results were expressed as percentages.

## 3. Results and Discussion

### 3.1. Results

#### 3.1.1. Sociodemographic Data

The study population included T2DM patients (*n* = 100), aged 35–70 years who were residing in Colombo District (Table 1), Sri Lanka.

#### 3.1.2. Awareness on Weight

Forty-four percent of the patients [females (*n* = 28) 50%; males (*n* = 16) 36%] were identified as either overweight or obese. However, only 60% of those patients accepted that they were either overweight or obese while 40% [*n* = 18; females, *n* = 23 (81.5%), males *n* = 8 (50%)] were reluctant to accept and consider a reduction in weight.

#### 3.1.3. Consumption of Main and Other Meals

Of the study population 98% consume all three meals on time and 34% ingest ≥5 small frequent meals/day as recommended by the Sri Lankan clinical practice guidelines for the management of diabetes [14]. High glycaemic index (GI) meals like bread [15], short eats, string hoppers [16],* pittu *(a steam cooked food made with rice flour and coconut), and so forth [17] are consumed by 17.8% for ≥5 days/week as breakfast while 8.9% consumed boiled legumes (low GI [17]) for breakfast. Consumption of rice meal as breakfast for all 7 days of the week was by 45.5%. Majority (72.2%) of the population consumes rice as breakfast for ≥4 days of the week.

All consumed rice meals for lunch which elicit a lower or medium glycaemic response irrespective of the rice variety [18], all days of the week except one. Consumption of high GI foods (white bread [15] and string hoppers [16], etc.) for dinner was by 13.3% (for 3 or more than 3 days/week) while only 4.4% relies on light diets (soup/soup with biscuits/biscuits with milk/small portion of a main meal/fresh fruits or vegetables, etc.) for ≥4 days/week. Rice consumption for dinner for all days of the week was by 45% and they consume rice for all three meals. From the population 83% consumed raw red rice (67%) or raw* nadu* (14%) or parboiled white/red rice (2%).

**Figure 1:**
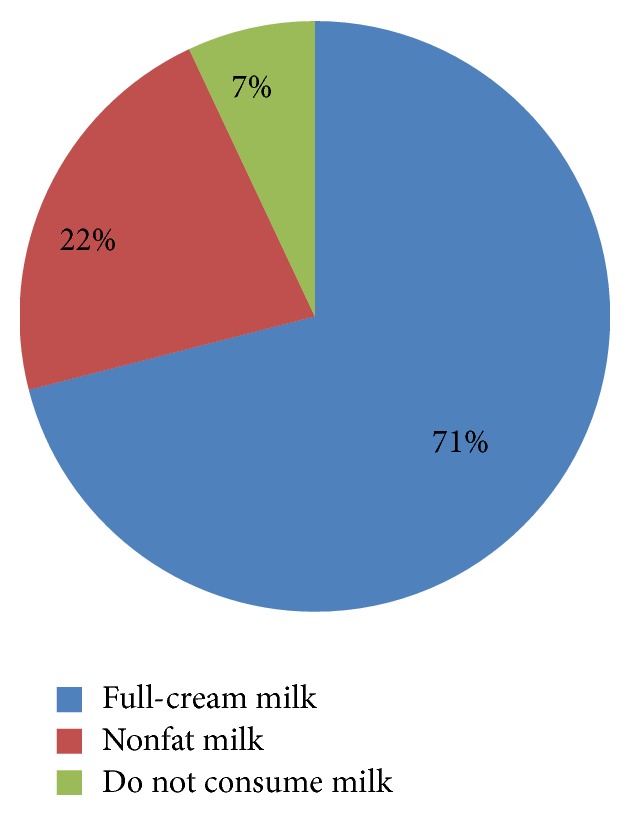
Milk consumption among patients with diabetes.

Only 67% consumed fruits at least once a day while 100% consumed vegetables for all three meals for all 7 days. When considering consumption of eggs, 30% of the population consumed egg/day for 2 or more than 2 days per week while 24% refrained from consuming eggs. Seventy percent of the study population consumed less than 2 eggs per week. The average chicken, pork, beef, and mutton consumption was 68%, 2%, 2%, and 1% at least once a week, respectively. A majority of the study population does not consume pork (87%), beef (93%), or mutton (92%). The consumption of chicken at least once a week was by 38%. All nonvegetarians (97%) consumed fish once or more than once a week. Fried foods/short eats and so forth, for 2 or more than 2 times/week, were consumed by 33% while 51% consumed them once or less than once per week. Full-cream milk is consumed by 71% and 22% consumed nonfat milk (Figure 1).

#### 3.1.4. Consumption of Green Leaves

From the study population, 99% consumed green leaves as* mallum* (mildly heated mixture of chopped green leaves and coconut scrapings in 2-3 portions : 1 portion), curry,* sambol *(chopped fresh leaves mixed with scraped coconut, green chillies, salt, and lime juice), and porridge. Sixty-four percent consumed green leaves daily and 35% consumed them 1–3 days/week. Sixty-seven percent (67%) of patients consumed leaves prepared by any method (*mallum*, curry,* sambol,* and porridge), while 33% consumed green leaves prepared by either one or two or three of the above methods. Herbal porridge consumption as part of the meal at least for 2-3 days per week was practiced by 23% and 41% consumed porridge 1–4 times/month while 33% did not consume porridges even though all herbal porridges elicit a low glycaemic response [19–21] and are rich in antioxidants [22].

Although the usage of green leaves as* sambol, mallum,* curry, or porridge is a long standing dietary practice, a change in the selection of the leaf varieties used was observed after diagnosis of diabetes in order to use leaves as a remedy by 58% of the study population.

**Figure 2:**
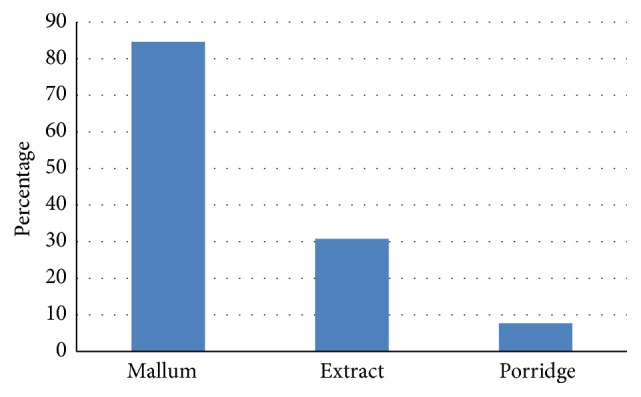
Consumption of green leaves as a remedy by diabetes patients.

*Sambol*,* mallum*, curry, extract (crude extract or tea), and porridge are used as herbal remedies and 58% of the population relies on herbal remedies in any form (Figure 2) and not due to prescription by ayurvedic physicians. Among them 96% consumed herbal remedies due to knowledge gained from other patients or folklore and only 4% relied on research data published on mass media. Among patients who relied on herbal remedies, majority (85%) consumed leaves as* sambol* or* mallum *or curry, 31% consumed them as an extract, and only 8% consumed them as porridge.

From the patients who rely on herbal remedies 75% consume* Cheilocostus speciosus* (thebu) as a salad or extract.* Scoparia dulcis* (walkoththamalli),* Nyctanthes arbor-tristis* (sepalika) flowers, and* Artocarpus heterophyllus* (kos) leaves are also commonly ingested as water extracts while* Cephalandra indica *(kowakka),* Murraya koenigii spreng *(karapincha), and* Adenanthera pavonina *(madatiya) are used as porridge. However, only 37% perceived a reduction of symptoms due to these remedies. None reported allergies to any leaf variety they consumed. Only 9% used other commercially available herbal products, infrequently, mainly green tea.

#### 3.1.5. Consumption of Sweetened Foods

Majority (99%) of the population consumed normal sweetened foods instead of low caloric/noncaloric foods. Consumption of any sweetened food or beverage once or more than once/day was by 3% of the population while 77% consumed any sweetened food or beverage once or less than once/week. However, none of the patients in the study used noncaloric sweeteners. Two-thirds (66%) of the population did not use sugar for tea and only 8% used more than 2 teaspoons of sugar for tea. The amount of sugar obtained from other foods was approximately less than 2 teaspoons/day for 56% of the population.

#### 3.1.6. Prevalence of Other Comorbidities and Diabetes Related Complications

**Figure 3:**
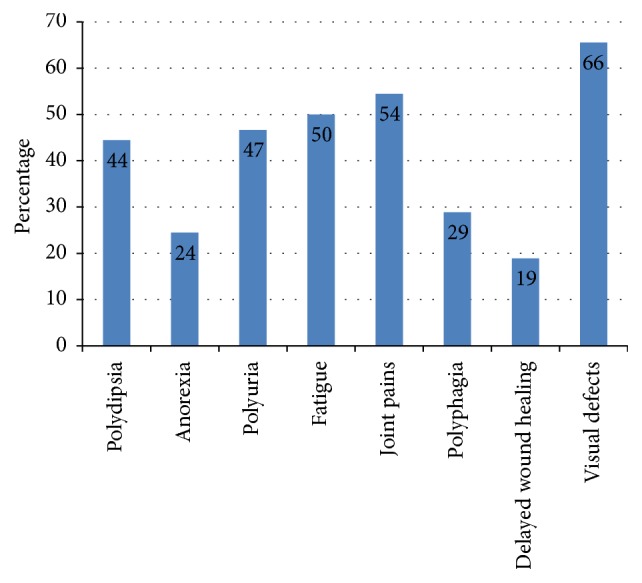
Comorbidities of diabetes patients.

Of the study population, 40%, 50%, 69%, and 11% had been diagnosed as hypertensive and hypercholesterolaemic and had visual impairment and heart diseases, respectively. According to the patients' perception, majority of the patients were suffering from visual impairment (66%) followed by joint pains (54%), fatigue (50%), increased frequency of urination (47%), polyphagia (29%), and loss of appetite (24%) (Figure 3) despite being on treatment.

#### 3.1.7. Physical Exercises

Only 14% of the study population exercised daily while 69% never exercised. Patients who exercised daily were also not following the recommended guidelines of time and activity level (jogging, brisk walking, dancing, gardening, etc.; 150 minutes per week). Majority (99%) of the patients was of the view that “day-to-day activities” (cooking, sweeping, shopping, washing, duty at work place, etc.) were sufficient exercises.

### 3.2. Discussion

Canadian diabetes association states that 80–90% of DM patients in the world are overweight or obese [23]. However, no recent data is available on the prevalence of overweight/obesity among diabetes patients in Sri Lanka. Among the overweight and obese subjects (44%) in the present study, 40% denied that they fell into either of these categories. Among the rest many were unaware whether their BMI is normal or not. This misperception on body weights was observed in a previous study as well. Nearly 66% and 44.7% of males and females who were overweight and over one-third of males and females who are obese considered themselves to be of “about right weight” [24]. The reason for overweight and obesity of Sri Lankans could be the lack of knowledge on proper diet control, consumption of highly refined carbohydrate containing foods which are conveniently available [7] prior to diagnosis of diabetes thus exceeding the recommendations [6], lack of physical exercise [6] even after diagnosis, and consumption of diets low in omega 3 fatty acids [25].

Consumption of 5 portions of fruits and vegetables/day is practiced only by 3.5% of healthy Sri Lankan population [6]. However, the present study with DM patients revealed this practice to be more prevalent among (45.5%) diabetes patients. As fatty food consumption of these patients was low, Sri Lankan diets with larger carbohydrate portions which exceed the upper limit of the recommendations for starch intake [6] and more importantly less exercise might be the contributory factors for overweight, obesity, and other diabetes complications among Sri Lankan diabetes patients. Rice mixed meals were the most consumed food among the study population. However, irrespective of rice mixed meals having a low GI if the rice portion is large the glycaemic load would be high and can lead to high glycaemic response [16]. Fish consumption among animal proteins was highest. Chicken was the meat more commonly consumed. Eggs were consumed by majority but within the recommendations (3-4 eggs/week). Thus the animal product consumption which will contribute to saturated fat and cholesterol intake could be a minor risk factor for the development of further complications in this population. Thirty-seven out of 50 participants, of a study conducted in 2015, were unaware of their protein intake and some were under the impression that they should discontinue meat consumption [9]. The practice of nonfat milk consumption was significantly low which was mainly due to less palatability.

Sweetened foods are responsible for an elevation of blood glucose. Persistent high blood glucose levels lead to the formation of advanced glycated end products responsible for diabetes related macrovascular and microvascular complications. Excess glucose is deposited as fat in adipose tissues causing overweight or obesity [26]. Therefore, limitation of sweetened foods or drinks consumption is a key step in the guidelines for the control of diabetes. Though the reported sugar consumption among Sri Lankans is significantly high [6] most tend to reduce sugar consumption after being diagnosed with diabetes. Thus, consumption of any sweetened food or beverage once or more than once a day was rare and only one-quarter reported to consume any sweetened food or beverage once or more than once a week. Accordingly the sugar intake of the diabetes patients in the present study was in accordance with the guidelines [14]. This “sugar” restriction could be due to the advice by health care professionals [9]. Seventy-two percent of diabetes patients in a study conducted in 2015 believed that diabetes arises due to high sugar intake during earlier stage in life and thus have completely avoided sugar consumption [9]. However, their knowledge on food types which elevate blood glucose concentration could be low, as some of them consume sugar alternatives (i.e., dates) in normal or even in higher amounts without restriction [9]. Noncaloric sweetener usage was not popular among the Sri Lankan diabetes patients. Most of them were unaware of the availability of noncaloric sweeteners, some were not satisfied with regard to safety and some disliked the taste. Educating patients regarding noncaloric sweeteners may further reduce the sugar consumption and increase the quality of life of diabetes patients.

A study revealed that an increase of one serving of green leafy vegetable consumption per day is associated with a modestly lower hazard of diabetes [27]. Consumption of leafy greens contributes to intake of certain vitamins and antioxidants and thus combats deficiencies that could arise with diabetes [28]. Green leaves made as* mallum*, curry,* sambol, *and porridge were consumed by the diabetes population. However, this study proved that one-third of Sri Lankan diabetes patients do not consume the recommended intake of green leafy vegetables (35%) which could contribute to reducing the carbohydrate intake and absorption.

Sri Lankan diabetes patients believe that certain foods such as fenugreek seeds* (uluhal)*,* Coscinium fenestratum *or yellow vine* (venivelgeta)*, curry leaves and powder, bitter-gourd, passion fruit leaves, finger millet, jack fruit leaves, wood apples,* Scoparia dulcis (walkoththamalli)* porridge, dried night jasmine flower,* Wattakaka volubilis *leaves* (anguna kola)*,* Costus speciosus (thebu)* leaves, and ceylon olive fruit* (veralu)* are effective in reducing blood glucose [9]. Ninety percent of Sri Lankan diabetes patients are reported to use one or more herbs as part of a meal or as a medication to control diabetes [29]. However, only 58% of the present study population consumed herbal remedies which could be due to the lack of availability of certain herbs in urban and suburban areas of Colombo District. Most of the patients tend to follow the remedies that the others follow in an ad hoc manner maybe due to the lack of knowledge on research data due to lack of accessibility to data sources. As some herbs cause toxic effects with long term consumption, patients need to be educated on the use and the dose of herbal remedies.

Although a previous study revealed that 60% of patients were knowledgeable that regular exercise could control blood glucose [30], only 14% of the present study population engaged in regular exercise [14]. Among the individuals who reported that they exercise, most considered “walking” for any other purpose also as exercising. Similar results were obtained in another study, which revealed that a considerable number of diabetes patients (18/50) were satisfied with their normal regular household work and considered these activities as “physical activities” [9].

This study also revealed that a significant number of the diabetes patients are not satisfied with their quality of life due to the symptoms that arise with the fluctuations of blood glucose which they could not control owing to lack of knowledge on diet management. A previous study indicated that the prevalence of neuropathy, nephropathy, retinopathy, coronary vascular disease, stroke, peripheral disease, and hypertension among newly diagnosed diabetes patients was 25.1%, 29%, 15%, 21%, 5.6%, 4.8%, and 23%, respectively [31]. As these complications aggravate with the duration of diabetes, patients should be educated to identify and to control these symptoms by diet, medicines, exercise, and frequent medical checkups. A recently carried out study revealed that although 70% of the Sri Lankan T2DM patients have a “good” or “very good” (score > 65) knowledge on diabetes compared to other South Asian countries more than 90% of patients could not recognize the symptoms of hypoglycaemia/hyperglycaemia [30].

Therefore, by improving the patients' knowledge on diabetes related signs and symptoms, diet control and self-management of diabetes could uplift their quality of life. Since this study was conducted at a single study center the results do not reflect the diet patterns of the general population and further studies are required to prove these outcomes.

## 4. Conclusion

Vegetables, fatty foods, and poultry consumption of diabetes patients of the present study were in accordance with the guidelines. Majority consumed full-cream milk and sugar intake was in accordance with the guidelines. The noncaloric sweetener usage was nonexistent and the majority was unaware of the availability of noncaloric sweeteners or foods made with noncaloric sweeteners. A significant percentage (45.5%) of diabetes patients consumed rice mixed meal for all three meals and consumption of five portions of fruits and vegetables/day was higher compared to the reported data of normal population. The daily green leafy vegetable intake and the quantity consumed were inadequate to obtain beneficial effects which can be correlated to the high rate of some complications and comorbidities. A considerable number of diabetes patients were unaware that they were either overweight or obese and some of the patients who were informed that they were obese or overweight were reluctant to accept the categorization. Also the patients were not aware of the wealth of scientific information available on many foods and herbal remedies.

## Figures and Tables

**Table 1:** Sociodemographic data.

Males	44
Females	56
Mean age	55 ± 9
Known as diabetes	
For less than 1 year	10%
For 1–10 years	56%
More than 10 years	34%
Attending diabetes clinic	72%
Monthly income	
<50,000 LKR	78%
50,000–100,000 LKR	18%
>100,000 LKR	4%
